# Disrupted lipid homeostasis as a pathogenic mechanism in *ABCA7*‐associated Alzheimer's disease risk

**DOI:** 10.1002/alz.71312

**Published:** 2026-03-30

**Authors:** Younji Nam, Brooke A. DeRosa, Aura M. Ramirez, Biniyam A. Ayele, Patrice Whitehead‐Gay, Larry D. Adams, Charles G. Golightly, Takiyah D. Starks, Mayra Juliana Laverde‐Paz, Holly N. Cukier, Rufus Akinyemi, Fred Sarfo, Albert Akpalu, Michael L. Cuccaro, Scott M. Williams, Allison Caban‐Holt, Christiane Reitz, Jonathan L. Haines, Goldie S. Byrd, Farid Rajabli, Derek M. Dykxhoorn, Juan I. Young, Jeffery M. Vance, Margaret A. Pericak‐Vance

**Affiliations:** ^1^ John P. Hussman Institute for Human Genomics University of Miami Miller School of Medicine Miami Florida USA; ^2^ Department of Neurology Addis Ababa University School of Medicine Addis Ababa Ethiopia; ^3^ Department of Social Sciences and Health Policy Wake Forest University School of Medicine Winston‐Salem North Carolina USA; ^4^ Department of Psychiatry and Behavioral Sciences Sylvester Comprehensive Cancer Center University of Miami Miller School of Medicine Miami Florida USA; ^5^ Dr. John T. Macdonald Foundation Department of Human Genetics University of Miami Miller School of Medicine Miami, Forida USA; ^6^ Neuroscience and Ageing Research Unit Institute for Advanced Medical Research & Training University of Ibadan College of Medicine Ibadan Nigeria; ^7^ Department of Medicine Kwame Nkrumah University of Science & Technology Kumasi Ghana; ^8^ Department of Medicine University of Ghana Medical School/Korle Bu Teaching Hospital Accra Ghana; ^9^ Department of Population & Quantitative Health Sciences School of Medicine Case Western Reserve University Cleveland Ohio USA; ^10^ Gertrude H. Sergievsky Center Taub Institute for Research on the Aging Brain Departments of Neurology, and Epidemiology Columbia University New York New York USA

**Keywords:** African ancestry, Alzheimer's disease, ATP binding cassette subfamily A member 7, frameshift deletion, lipid droplet

## Abstract

**INTRODUCTION:**

*ABCA7* (ATP binding cassette subfamily A member 7) encodes a lipid transporter associated with increasing risk for Alzheimer's disease (AD). A 44‐base pair deletion in *ABCA7* (rs142076058; p.Arg578Alafs) is a strong risk factor in individuals of African ancestry (AA). However, the biological consequences of this deletion are poorly understood.

**METHODS:**

We expressed the truncated ABCA7 protein in HEK and HepG2 cells to assess cellular localization and impact on lipid metabolism, respectively. Additionally, induced pluripotent stem cell (iPSC)‐derived neurons carrying the deletion were functionally assessed compared to isogenic controls.

**RESULTS:**

Truncated ABCA7 localized to endoplasmic reticulum and plasma membranes similarly to the wild type in HEK cells but induced significant lipid droplet accumulation in HepG2 cells and iPSC‐derived neurons while reducing mitochondrial membrane potential in iPSC‐derived neurons.

**DISCUSSION:**

These findings show that the AA‐specific *ABCA7* deletion disrupts lipid and mitochondrial homeostasis, supporting a mechanistic link between the *ABCA7* deletion and increased AD risk.

## Highlights

- Truncated ATP binding cassette subfamily A member 7 (ABCA7) remains stable and localizes to endoplasmic reticulum and plasma membranes in HEK cells.
- Truncated ABCA7 disrupts lipid droplet regulation in HepG2 cells and neurons.
- *ABCA7* shows the highest expression in neurons among brain cell types.
- *ABCA7* truncation impairs lipid metabolism and mitochondrial health in neurons.

## 1 INTRODUCTION

Alzheimer's disease (AD) is the most common form of dementia, accounting for ≈ 65% cases.^1^ Pathologically, AD is characterized by extracellular amyloid beta (Aβ) plaques and intracellular neurofibrillary tangles.^2^, ^3^ Additionally, early alteration in neuroinflammation with microglial and astrocytic activation, synaptic dysfunction, cerebrovascular changes, and mitochondrial and lipid metabolic dysfunctions have been described in AD.^4^, ^5^, ^6^ While age is the strongest risk factor for AD, extensive evidence demonstrates that genetic factors also play a critical role.^6^, ^7^, ^8^, ^9^ The most well‐established genetic risk factor is the ε4 allele of apolipoprotein E (*APOE*).^10^ apoE is essential for lipid transport and metabolism in the brain, processes that maintain neuronal function and integrity.^11^, ^12^, ^13^ Disrupted lipid transport impairs membrane repair, synaptic signaling, and promotes Aβ accumulation.^14^

Individuals of African ancestry (AA) are nearly twice as likely to develop AD compared to individuals of European ancestry, yet *APOE* ε4 shows lower risk associations in African and sub‐Saharan African populations.^15^, ^16^ This suggests that additional ancestry‐specific risk factors may influence AD susceptibility. One such factor is ATP binding cassette subfamily A member 7 (*ABCA7*), a gene implicated in lipid homeostasis, phagocytosis, and Aβ clearance.^17^, ^18^ Indeed, recent studies have shown that ABCA7 binds to *APOE* and is involved in the lipidation of apoE.^17^, ^19^ While common *ABCA7* variants are associated with AD in European populations, rare and population‐specific variants show stronger effects in African descent populations.^20^

**FIGURE 1 alz71312-fig-0001:**
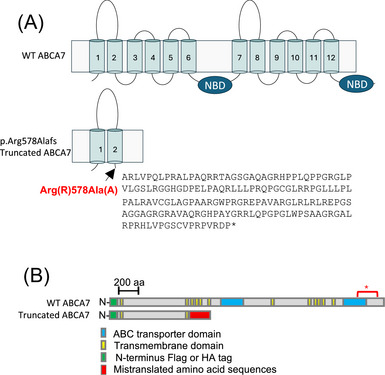
Scheme of WT and the predicted truncated ABCA7 proteins. A, WT ABCA7 has 12 hydrophobic TMDs and two NBDs. The deletion is predicted to result in an Arg578Alafs*171 alteration, which would truncate >70% of the protein and add 171 incorrect amino acids. The predicted truncated ABCA7 contains the first two TMDs without any NBDs. The mistranslated amino sequences are predicted to have no function. B, A schematic diagram of the N‐terminal Flag‐ or HA‐tagged WT *ABCA7* and truncated ABCA7. The red star indicates the localization of the epitope for commercially available C‐terminal ABCA7 antibodies. ABCA7, ATP binding cassette subfamily A member 7; NBD, nucleotide‐binding domain; TMD, transmembrane domain; WT, wild type.

Unique to the African ancestry, we identified a 44‐base pair (bp) deletion (rs142076058) in *ABCA7* that is significantly associated with AD.^20^ This *ABCA7* deletion produces a frameshift mutation (p.Arg578Alafs) predicted to encode a truncated protein containing only 2 of the 12 transmembrane domains and lacking both nucleotide‐binding domains (Figure 1A).^20^

Despite its strong genetic association with AD in AA populations, the functional impact of this *ABCA7* deletion remains poorly understood. Several factors make this mutation an excellent candidate to study the role of *ABCA7* in increased AD risk. Our previous demonstration that the mutated mRNA escapes nonsense‐mediated decay (NMD) indicates that the variant likely produces a truncated protein rather than complete loss of expression.^20^ This suggests that its pathogenicity is more likely due to loss of normal ABCA7 function or gain of function rather than haploinsufficiency due to transcript degradation. Given ABCA7's established role in lipid transport, endolysosomal function, and Aβ clearance, the presence of a truncated protein resulting from this deletion could plausibly amplify AD‐related pathology. Indeed, this truncated protein would be predicted to interfere with one of the phosphoserine lipid binding sites in the first transmembrane domain of ABCA7,^17^, ^19^ which are thought to be involved in lipidation of apoE. Further, our recent genetic interaction studies demonstrate that *APOE* ε3/ε4 carriers who also harbor the *ABCA7* 44‐bp deletion exhibit a significantly earlier age of AD onset compared to ε3/ε4 carriers lacking the deletion.^21^ This finding supports a potential synergistic interaction between *APOE* ε4 and the *ABCA7* deletion in accelerating disease progression. Finally, the incomplete penetrance of the deletion on AD development^20^ indicates potential involvement of additional genetic modifiers, environmental influences, or compensatory biological mechanisms. These observations underscore the need to more precisely define the molecular and cellular impact of the *ABCA7* deletion on AD.

**RESEARCH IN CONTEXT**

1. **Systematic review**: We conducted a systematic review to assess the functional impact of ATP binding cassette subfamily A member 7 (*ABCA7*) variants in Alzheimer's disease (AD). While AD research has focused on risk factors such as apolipoprotein E (*APOE*) ε4 and amyloid beta pathology, recent studies emphasize ancestry‐specific variants in African populations. A 44‐base pair deletion in *ABCA7* (rs142076058) has been associated with increased AD risk in individuals of African ancestry, but its functional role remains unclear.
2. **Interpretation**: Our results show that the truncated ABCA7 protein is stable and disrupts cellular lipid metabolism and mitochondrial function, suggesting interference with key biological pathways involved in AD development and progression.
3. **Future directions**: The *ABCA7* deletion may exacerbate lipid transport deficits already observed in *APOE* ε4 carriers, potentially contributing to an earlier age of AD onset. Further studies exploring the functional interplay between *ABCA7* deletions and *APOE* alleles are needed to elucidate this relationship and shed light on the underlying pathological mechanisms of *APOE* ε4.

Therefore, we conducted[Fig alz71312-fig-0001] functional studies to investigate the molecular and cellular consequences of the AA‐specific 44‐bp *ABCA7* deletion. These experiments aim to clarify how this variant disrupts *ABCA7* function and contributes to AD pathogenesis, ultimately improving our understanding of ancestry‐specific genetic risk and guiding the development of more equitable precision medicine strategies. Herein, we demonstrate that the truncated ABCA7 protein is stable, properly localized, and functionally alters cellular lipid metabolism and mitochondrial health. These results support a mechanism that may disrupt key processes in AD pathogenesis and provide novel insight into the functional role of *ABCA7* in ancestry‐specific genetic variation, highlighting potential therapeutic avenues, particularly for historically underrepresented populations.

## 2 METHODS

### 2.1 ABCA7 Flag‐ or HA‐ tagged plasmid construction

A wild‐type *ABCA7* plasmid construct with the human transcript NM_019112 and Flag‐tagged on the N‐terminus (EX‐A3083‐M11) was obtained from GeneCopoeia (Rockville). To mimic the 44‐bp deletion (rs142076058), a 308 bp DNA fragment spanning the deletion was polymerase chain reaction (PCR) amplified from a custom‐made pUC57 plasmid (44‐bp del pUC57 Plasmid – Cat No: SC1691, GenScript). The 44‐bp deletion pUC57 plasmid was digested with FseI and BstEII (New England Biolabs), and the smaller, 296‐bp fragment including the region with the deletion was purified from a 0.8% agarose gel using a QIAquick gel extraction kit (Qiagen). The GeneCopoeia plasmid was also digested with FseI and BstEII and treated with calf intestinal alkaline phosphatase (CIP; New England Biolabs) for 30 minutes at 37^°^C. The fragment with the deletion was then ligated into the GeneCopoeia backbone overnight with T4 DNA ligase. Sanger sequencing with the following primers (forward: CTTCGTGTACCTGCAAGACC, reverse: GCTGAGCAGGAAGCTCTG) confirmed that the 44‐bp deletion was successfully included in the GeneCopoeia Flag‐tagged plasmid using our previously published methods.^20^ For HA tags, the Flag tag in the Flag wild type (WT) *ABCA7* and Flag‐del *ABCA7* constructs was replaced with an HA tag for co‐localization studies. This HA tag replacement was achieved using the In‐Fusion Snap Assembly Kit (Cat# 638945, Takara), following the manufacturer's instructions. Primers encoding the HA sequence (forward: ATACGATGTTCCAGATTACGCTGAAGGAACAAGTTTGTACAAAAAAG; reverse: TCTGGAACATCGTATGGGTACATGGTCCGCGGCCTAGG) were designed using the primer design tool provided by the manufacturer for PCR‐mediated tag replacement. PCR‐amplified products were treated with DpnI to eliminate parental Flag‐tagged templates and subsequently purified using the NucleoSpin Gel and PCR Clean‐up Kit (Cat# 638945, Takara). The linearized PCR products were then circularized, transformed into competent cells, and plasmid DNA was isolated. Successful incorporation of the HA tag was confirmed by Sanger sequencing.

### 2.2 Immortalized cell lines, cell culture, transfection

HEK‐APPsw cells are modified from HEK293 to express the Swedish mutant amyloid precursor protein. In this study, HEK‐APPsw cells were used as an equivalent model to HEK293 cells and were a gift from Dr. Peter St. George‐Hyslop. HepG2 cells were purchased from ATCC (Cat#: HB‐8065). Cell lines were routinely maintained in Dulbecco's Modified Eagle Medium/Nutrient Mixture F‐12 (DMEM/F12, Cat#: 21331020, Gibco), supplemented with 10% fetal bovine serum (FBS, Cat#: A5670701, Gibco) and 1% penicillin–streptomycin (P/S, Cat#: 15140122, Gibco) at 37°C in a humidified 5% CO2 atmosphere. *cDNA3.1* (empty vector transfection control), tagged‐*ABCA7* WT, or tagged‐truncated *ABCA7* plasmids were transfected into HEK and HepG2 cells using the JetPRIME or JetOPTIMUS reagent (Cat#: 101000027 or 101000006, Sartorius) according to the manufacturer's protocol. Briefly, cells were seeded 24 to 48 hours before transfection. At ≈ 70% confluency, the transfection solution containing DNA was added to the cells dropwise. The cells were incubated overnight for post‐transfection treatments. The transfection with these vectors resulted in high transfection efficiency (> 90%) in HEK cells, but lower in HepG2 cells (≈ 30%) and iNeurons (> 0.01%).

### 2.3 Western blotting (WB)

Phosphate‐buffered saline (PBS)‐washed cells were lysed with ice‐cold radioimmunoprecipitation assay buffer (Cat#: 89900, Thermo Fisher Scientific) containing 1X protease inhibitor cocktail (Cat#: P8340, Sigma‐Aldrich) on ice for 15 minutes. The lysates were then centrifuged at 14,000 g for 10 minutes at 4°C to separate the protein (supernatant) from cellular debris (pellet). The protein concentration of the supernatant was measured using a bicinchoninic acid (BCA) protein assay kit (Cat#: 23225, Thermo Fisher Scientific). Fifteen micrograms of BCA‐measured cell lysates, prepared without boiling, were resolved using sodium dodecyl sulfate polyacrylamide gel electrophoresis on 3% to 8% gradient Tris‐acetate gels (Cat#: EA03752BOX, Thermo Fisher Scientific) and blotted onto 0.45 µm polyvinylidene fluoride membrane (Cat#: LC2005, Thermo Fisher Scientific) using the Trans‐Blot Turbo Transfer System. Membranes were blocked with a 5% final concentration of non‐fat dry milk powder for 1 hour. The membranes were probed with primary antibodies, including anti‐ABCA7 (Cat#: SC377335, SantaCruz), anti‐Flag (Cat#: F1804, Sigma‐Aldrich), and anti‐Actin (Cat#: AB179467, abcam) overnight at 4°C, followed by probing with secondary antibodies, including anti‐mouse and anti‐rabbit immunoglobulin G1 horseradish peroxidase for 1 hour.

### 2.4 Cellular fractionation into the membrane and cytoplasm

Subcellular fractionation was performed using a Subcellular Protein Fractionation Kit for Cultured Cells (Cat# 78840, Thermo Fisher Scientific) according to the manufacturer's protocol. Briefly, transfected HEK cells (2 × 10^6^ cells) were treated with cytoplasmic, membrane, and nuclear extraction buffers containing a protease inhibitor (included in the kit). In each step, subcellular components were collected by centrifugation and used for WB analysis.

### 2.5 Culture and generation of human isogenic induced pluripotent stem cells

Parental induced pluripotent stem cell (iPSC) lines for Sets A and B were generated in house using our established reprogramming protocol. Pluripotency and Sendai virus clearance were verified for each line (Figure S1A and B in supporting information). The parental iPSC line used for Set C (ADRC iPS 80, Clone 4) was obtained from the Induced Pluripotent Stem Cell Core, Alzheimer's Disease Research Center, University of California Irvine Institute for Memory Impairments and Neurological Disorders. iPSC lines containing specific knock‐in (KI) variants were generated using CRISPR/Cas9‐mediated homology‐directed repair. Single guide RNAs (sgRNAs) were synthesized by Synthego, reconstituted in nuclease‐free 1× TE buffer at 100 µM, pulse vortexed, and incubated on ice for 30 to 60 minutes before aliquoting and storing at −80°C. To form ribonucleoprotein (RNP) complexes, 140 pmol of sgRNA was combined with 40 pmol of Cas9 nuclease (EnGen Spy Cas9 NLS, Cat# M0646T, New England Biolabs) in Lonza P3 Nucleofector solution (Cat# V4XP‐3032) at a 3.5:1 molar ratio, then incubated at room temperature (RT) for 10 minutes. iPSCs were cultured in mTeSR Plus medium (StemCell Technologies) on vitronectin‐coated plates (Cat# A14700, Thermo Fisher Scientific) and pre‐treated for at least 1 hour with 10 µM Y27632 and 2 µM thiazovivin to improve cell viability. After dissociation with Accutase, cells were washed in DMEM/F12 (Cat# 10565042, Thermo Fisher Scientific) and resuspended in nucleofection solution containing 3 µM donor single‐stranded oligodeoxynucleotide (ssODN; IDT Ultramer, 100 µM stock). A total of 2 × 10^5^ cells were mixed with the RNP complexes and electroporated using the 4D‐Nucleofector X Unit (Lonza) with program CA137. Transfected cells were plated onto vitronectin‐coated 12‐well plates and cultured in mTeSR Plus supplemented with 10 µM Y27632 and 2 µM thiazovivin. After recovery, bulk‐transfected cells were cryopreserved, and genomic DNA was extracted from cell pellets using QuickExtract DNA Extraction Solution (Cat# QE09050, Qiagen). Editing efficiency was evaluated through Sanger sequencing and analyzed with the ICE tool (Synthego). To isolate single‐cell clones, limiting dilution was performed at 1 cell per 100 µL in mTeSR Plus with CloneR2 (StemCell Technologies), plated onto rhLaminin‐521–coated 96‐well plates (Cat# A29248, Thermo Fisher Scientific). After 7 to 10 days, emerging colonies were visually screened, cryopreserved in CryoStor CS10 (StemCell Technologies), and genotyped. Selected clones were thawed into mTeSR Plus supplemented with CloneR2 (Cat#: 100‐0691, StemCell), expanded, and verified by Sanger sequencing to confirm successful KI incorporation.

During the generation of CRISPR‐edited lines, efforts to obtain homozygous or heterozygous clones carrying a 44‐bp deletion in *ABCA7* were challenging, yielding either no clones or a very small number (< 3%), none of which survived the iPSC expansion process (Figure S2A in supporting information). However, a higher number of clones (> 10%) with a 47‐bp homozygous or heterozygous deletion in *ABCA7* were successfully obtained. Although the underlying mechanism is unclear, potentially, the homology at the site adjacent to the deletion might have influenced the DNA repair machinery, favoring the formation of a 47‐bp deletion over the 44‐bp deletion. Because the 47‐bp deletion removed an additional single codon (Leu[L]577Ala[A]), minimally altering the resulting protein compared to the 44‐bp deletion (Arg[R]578Ala[A]; Figure S2B and C), the 47‐bp deletion clones were selected as functional equivalents of the 44‐bp deletion in *ABCA7*. Genomic stability of the isogenic iPSC lines was assessed by G‐banding karyotyping (Figure S3 in supporting information). Additionally, Sanger sequencing was performed to confirm the genotypes and assess the top five potential CRISPR off‐target effects in the *ABCA7* deletion lines, as predicted by the CRISPOR tool.^22^ CRISPR‐generated isogenic *ABCA7* deletion iPSC lines were differentiated into neurons by a conventional neuronal induction approach, using a transdifferentiation protocol to overexpress Neurogenin‐2 (*NGN2*) using home‐brew cocktails. Throughout neuronal induction and maturation, no significant developmental defects were observed in neuronal structures (Figure S4 in supporting information).

### 2.6 Transdifferentiation to iPSC‐derived neurons (iNeurons)

PB‐NGN2‐EF1a‐Puro‐BFP and PB‐transpose plasmids were gifted by Dr. Samuele Marro from the Nash Family Department of Neuroscience and the Black Family Stem Cell Institute at the Icahn School of Medicine at Mount Sinai. The generation of iNeurons from human iPSCs was described previously.^23^, ^24^ Briefly, iPSC cultures at 70% to 80% confluency were visually inspected to ensure proper morphology and the absence of untargeted differentiation. On day 0, 3 × 10^4^ single‐cell dissociated iPSCs were seeded onto a 1w/6w plate in mTeSR+ (Cat#: 100‐0276, StemCell). On day 1, cells were transfected with PB‐NGN2‐EF1a‐Puro‐BFP and PB‐transpose plasmids using Lipofectamine Stem Transfection Reagent (Cat#: STEM00015, Thermo Fisher Scientific) according to the manufacturer's protocol. After 4 hours of transfection, 2 mL of mTeSR^+^ was added, and the cells were incubated overnight. On day 2, the media was aspirated and replaced with fresh 1.5 mL mTeSR^+^. On days 3 and 5, transfection selection was initiated by adding puromycin (final concentration of 2 µg/mL, Cat#: 540411, Sigma‐Aldrich). On day 6, cells were passed onto a 6w/6w plate for full puromycin selection. On days 7 and 9, cells were continued on mTeSR+ with puromycin. By days 11 to 13, when the confluency reached > 70%, integration of PB‐ *NGN2*‐EF1a‐Puro‐BFP was confirmed (> 95% BFP expression), and neuronal induction and maturation were initiated. On day 0, 1 × 10^6^ single‐cell dissociated cells were seeded onto a Matrigel‐coated 1w/6w plate in a home‐brewed neuronal induction media^23^ containing 2 µg/mL doxycycline with CultureCEPT (Cat#: A56799, Thermo Fisher Scientific). On days 1 and 2, cells were washed and replaced with induction media without CultureCEPT. On day 3, cells were treated with 1 µM uridine (Cat#: U3003, Sigma‐Aldrich) and fluorodeoxyuridine (Cat#: F0503, Sigma‐Aldrich) to suppress mitotic events. On day 4, cells were replated (1–2 × 10^6^ cells) onto a PLO‐coated 1w/6w plate in home‐brewed neuronal maturation media I^23^ with CultureCEPT. On day 5, the media was replaced without CultureCEPT. On day 8, cells were fed with a ½ media change. On days 11, 13, 17, 23, and 26, cells were fed with home‐brewed neuronal maturation media II.^23^ On day 28, mature iNeurons were harvested for the desired assays. For aged iNeurons, cells were additionally aged for 60 days and used for the mitochondria health assay.

### 2.7 Immunocytochemistry

Cells were fixed in 4% methanol‐free formaldehyde (Image‐iT Fixative Solutions, Cat#: I28800, Thermo Fisher Scientific) at RT for 15 minutes, washed three times with PBS, then blocked/permeabilized with 10% Normal Donkey Serum (Cat#: 017‐000‐121, Jackson ImmunoResearch), 0.25% Triton X‐100, and 0.05% Tween‐20 for 1 hour. Cells were then incubated with primary antibodies, including anti‐ABCA7 (Cat#: SC377335, SantaCruz), anti‐Flag (Cat#: F1804, Sigma‐Aldrich), anti‐β‐Tub III (Cat#: AB78078, abcam), and anti‐MAP2 (Cat#: 13‐1500, Thermo Fisher Scientific), in a blocking solution overnight at 4°C or for 1 hour at RT. Cells were washed three times with PBS, then incubated with secondary antibodies in a blocking buffer for 1 hour at RT. Cells were incubated with Hoechst 33342 (Cat#: R37605, Thermo Fisher Scientific) for 15 minutes at RT, followed by 3X PBS washes for 5 minutes. Cells were washed once more and replenished with PBS for imaging.

### 2.8 Treatments and lipid droplet assay

Transfected HepG2 cells with WT *ABCA7*, del *ABCA7*, or empty *pcDNA3.1* plasmids were treated with either 300 µM bovine serum albumin (BSA)–oleic acid (OA, Cat#: 29557, Cayman) to induce lipid droplet (LD) accumulation or control BSA (Cat#: 34932, Cayman) overnight. LDs were visualized using LipidSpot 610 or 488 dye (70069 or 70065, Biotium) for 30 minutes in live cells, followed by fixation and subsequent immunofluorescence staining. The accuracy of LipidSpot in detecting LDs was evaluated by comparing it to conventional Nile Red staining (Cat# N1142, Invitrogen) in HepG2 cells subjected to 300 nM BSA or OA for co‐localization immunocytochemistry (ICC) analysis (Figure S5 in supporting information). Only cells that showed a positive signal for expression of the recombinant version of ABCA7 (as determined by immunocytochemical staining for the N‐terminal Flag tag) were assessed for the determination of LD formation.

### 2.9 Mitochondrial health assay

iNeurons were aged for an additional 32 days in culture, resulting in a total culture duration of 60 days. The cells were fixed and processed for assessment of mitochondrial health. Staining was performed using the HCS Mitochondrial Health Kit (Cat# H10295, Invitrogen) according to the manufacturer's instructions. Briefly, the assay evaluates mitochondrial membrane potential and overall mitochondrial function, allowing quantification of active versus depolarized mitochondria.

### 2.10 Light microscopy image acquisition and analysis

Images were acquired using either a confocal microscope (LSM980, Zeiss) with Airyscan 2 or an automated high‐resolution fluorescence microscope (BZ‐X800, Keyence) equipped with DAPI, Green, Texas Red, and FarRed channels. Acquisition parameters, including exposure time and image quality for each channel, were maintained consistently across all samples to minimize bias. For LD quantification, representative cells were cropped to 1‐inch squares. Individual cell boundaries were manually delineated in ImageJ, and images were converted to 8‐bit for thresholding. Signals outside the defined cell borders were manually excluded using the paintbrush tool, and thresholding was applied to calculate the percentage of lipid area per cell.

Colocalization of protein expression between two channels was quantified using ImageJ with the Coloc 2 plugin. Analysis was performed on the entire cell image, without drawing regions of interest or applying binary masks. No intensity threshold was applied, so all pixel values were included. Colocalization was quantified using the Pearson correlation coefficient (*R*), which measures the linear correlation of pixel intensities between the two channels. Pearson *R* values range from –1 (complete anti‐correlation) to 1 (perfect correlation), with higher positive values indicating a greater degree of colocalization. Analysis was performed on the entire cell image without drawing regions of interest or applying binary masks. No intensity threshold was applied; all pixel values were included.

### 2.11 Statistical analysis

All statistical analyses were performed using JMP Pro 17 (SAS Institute Inc.). Comparisons between groups were conducted using two‐tailed Student *t* tests. A *P* value of < 0.05 was considered statistically significant. Post hoc power analysis was performed using Tukey post hoc analysis.

## 3 RESULTS

### 3.1 The truncated ABCA7 protein is stable and is localized to the endoplasmic reticulum and plasma membrane in HEK cells

**FIGURE 2:**
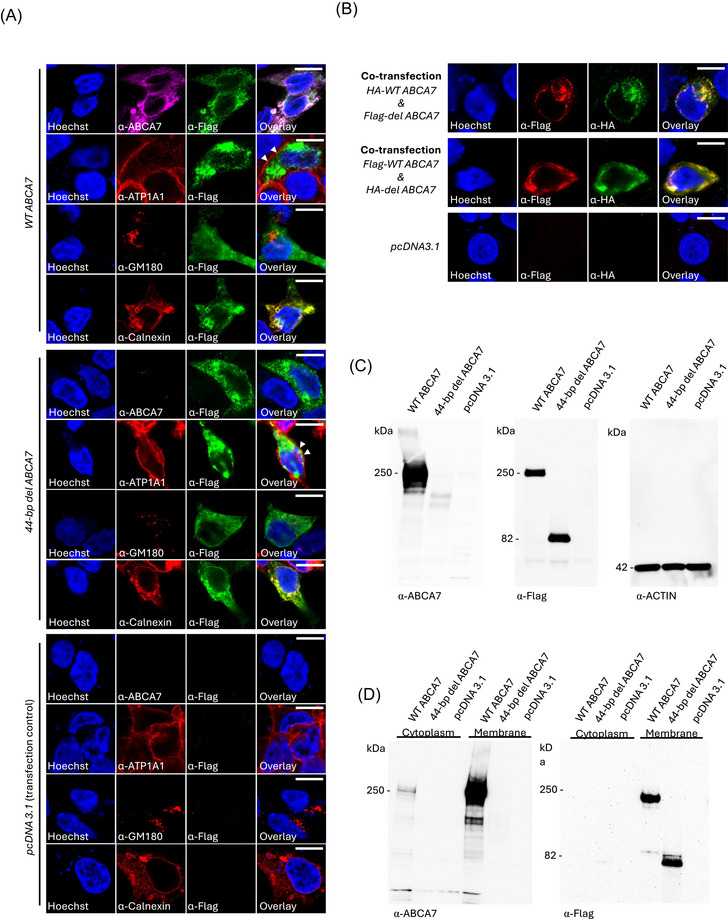
Truncated ABCA7 is stable and localizes at the ER membrane and plasma membrane. A, ICC of HEK cells transfected with either WT *ABCA7*, truncated *ABCA7*, or *pcDNA3.1* (empty vector, transfection control) probed with Hoechst (nucleus marker), anti‐Flag, anti‐ABCA7 antibodies, anti‐ATP1A1 (plasma membrane marker), anti‐GSM180 (Golgi surface membrane marker), and/or anti‐Calnexin (ER membrane marker). B, ICC of HEK cells co‐transfected with HA‐WT *ABCA7* and Flag‐del *ABCA7* or Flag‐WT *ABCA7* and HA‐del *ABCA7* probed with Hoechst (nucleus marker), anti‐HA, and/or anti‐Flag. C, Western blot analysis of transfected HEK cells with either anti‐ABCA7, anti‐Flag, or anti‐ACTIN as a loading control. Confocal images were collected on a ZEISS LSM 980 equipped with Airyscan 2 using a 63× oil‐immersion objective and 5× optical zoom. Scale bar, 10 µm. E, Western blot analysis of transfected HEK cells subjected to subcellular fractionation (cytoplasm and membrane) with either anti‐ABCA7 or anti‐Flag. ABCA7, ATP binding cassette subfamily A member 7; ER, endoplasmic reticulum; ICC, immunocytochemistry; WT, wild type.

To investigate whether the 44‐bp del *ABCA7* affects protein stability and localization, we transfected HEK cells with a plasmid encoding either the N‐terminally Flag‐tagged full‐length (wild‐type, WT *ABCA7*) or the truncated ABCA7 predicted to result from the 44‐bp deletion (Figure 2A). ICC analysis for Flag immunodetection showed that both the full‐length and truncated ABCA7 were localized predominantly at the endoplasmic reticulum (ER) membrane (co‐localization with Calnexin) with some accumulation at the plasma membrane (co‐localization with ATP1A1, white triangles; Figure 2A). As expected, an antibody against the C‐terminal domain of ABCA7 detected WT ABCA7 but not the truncated ABCA7 protein lacking the C‐terminus. To further confirm that truncated ABCA7 localization does not differ from its WT counterpart, we co‐transfected an HA‐tagged WT *ABCA7* version along with a Flag‐tagged del *ABCA7* version, and vice versa (Figure 2B). The signals for both the WT and truncated versions of ABCA7 overlapped in these cells. Similar overlapping expression patterns of both WT and truncated ABCA7 were also observed in transfected HepG2 cells and early iNeurons (Figure S6A and B in supporting information). To quantify the subcellular localization of WT ABCA7 and truncated ABCA7, we performed colocalization analysis using Pearson correlation coefficient (*R*) in cells co‐transfected with both constructs. In HEK cells (*n* = 6), Pearson *R* values were 0.87, 0.94, 0.95, 0.94, 0.95, and 0.93, indicating a high degree of colocalization. High colocalization was also observed in HepG2 cells (*R* = 0.97) and in iNeurons (*R* = 0.87). These results indicate that WT ABCA7 and truncated largely occupy the same subcellular compartments across multiple cell types. This suggests that, in at least this case, ABCA7 mislocalization is not the driver of the AD‐associated pathogenic mechanism(s).

To confirm that the truncated version of ABCA7 was stable, immunoblot analysis was performed on the transfected HEK cells. Immunoblotting using an anti‐Flag antibody showed similar levels of WT ABCA7 and truncated ABCA7, with each migrating at its expected sizes (≈ 240 kDa for WT and 82 kDa for the truncated version; Figures 2C and S7A in supporting information). Additionally, subcellular fractionation was performed to separate cytoplasmic and membrane fractions from the transfected cells (Figures 2D and S7B). The relative distribution of the protein was quantified by calculating the percentage of band intensity present in each fraction (Figure S7C). Both WT ABCA7 and truncated ABCA7 localized to the membrane fraction, confirming the results of the immunocytochemical analysis. Together, these findings indicate that the truncated ABCA7 protein is stable, correctly transported, and localized to the ER membrane and plasma membrane in transfected HEK cells (and HepG2 cells).

### 3.2 The truncated ABCA7 protein affects lipid droplet formation in HepG2 cells

**FIGURE 3:**
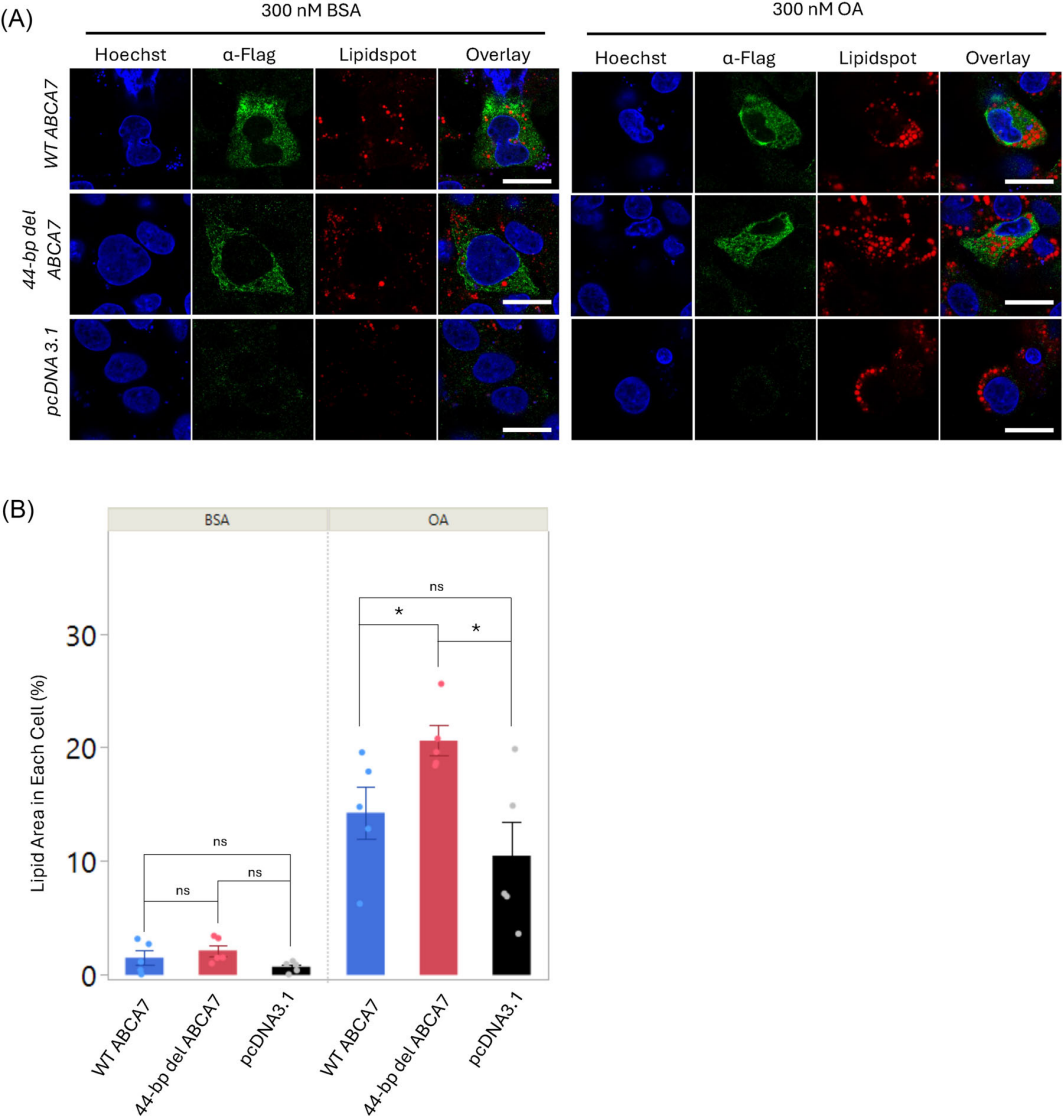
Functional effect in lipid droplet metabolism of truncated ABCA7 in transfected HepG2 cells. A, Images of HepG2 cells transfected with WT *ABCA7*, truncated *ABCA7*, *pcDNA3.1* (empty vector, transfection control), stained for the detection of Flag and LDs in the presence of 300 nM BSA (left three panels) or OA (right three panels). Confocal images were collected on a ZEISS LSM 980 equipped with Airyscan 2 using a 63× oil‐immersion objective and 3× optical zoom. Scale bar, 16.7 µm. B, Quantitation of LD area (%) per transfected cell in the presence of 300 nM BSA (left three bars) or OA (right three bars) shows that the effect of the ABCA7 truncated protein is different than the WT. These experiments were performed twice independently with technical triplicates. The dots overlaid on each bar represent the individual data points. Data represent the mean ± standard error of the mean, *P < 0.05, ns, non‐significant (one‐way analysis of variance followed by a Tukey *t* post hoc test). ABCA7, ATP binding cassette subfamily A member 7; BSA, bovine serum albumin; LD, lipid droplet; OA, oleic acid; WT, wild type.

To test if truncated ABCA7 could influence LD accumulation, we transfected HepG2 cells with either WT or deletion *ABCA7* and compared LD accumulation by staining with LipidSpot. We used the liver‐derived HepG2 cells, which exhibit LD formation when exposed to fatty acids like oleic and palmitic acid.^25^ Minimal LD accumulation was seen in HepG2 cells exposed to BSA (vehicle control), and this basal level of lipid droplets was not affected by overexpression of either the WT or deletion *ABCA7* (Figure 3A). However, OA exposure led to a pronounced increase in LD accumulation across transfected HepG2 cells. Although the WT ABCA7–expressing cells showed a modest increase in LD compared to the empty vector control cells, this difference was not statistically significant (Figure 3A and B). On the other hand, the cells expressing the truncated ABCA7 showed a significant increase in LD accumulation compared to both the empty vector and, importantly, the WT ABCA7‐expressing cells (Figure 3A and B). These findings suggest that truncated ABCA7 plays a role in regulating LD accumulation in HepG2 cells.

### 3.3 *ABCA7* is expressed at the highest level in neurons

**FIGURE 4:**
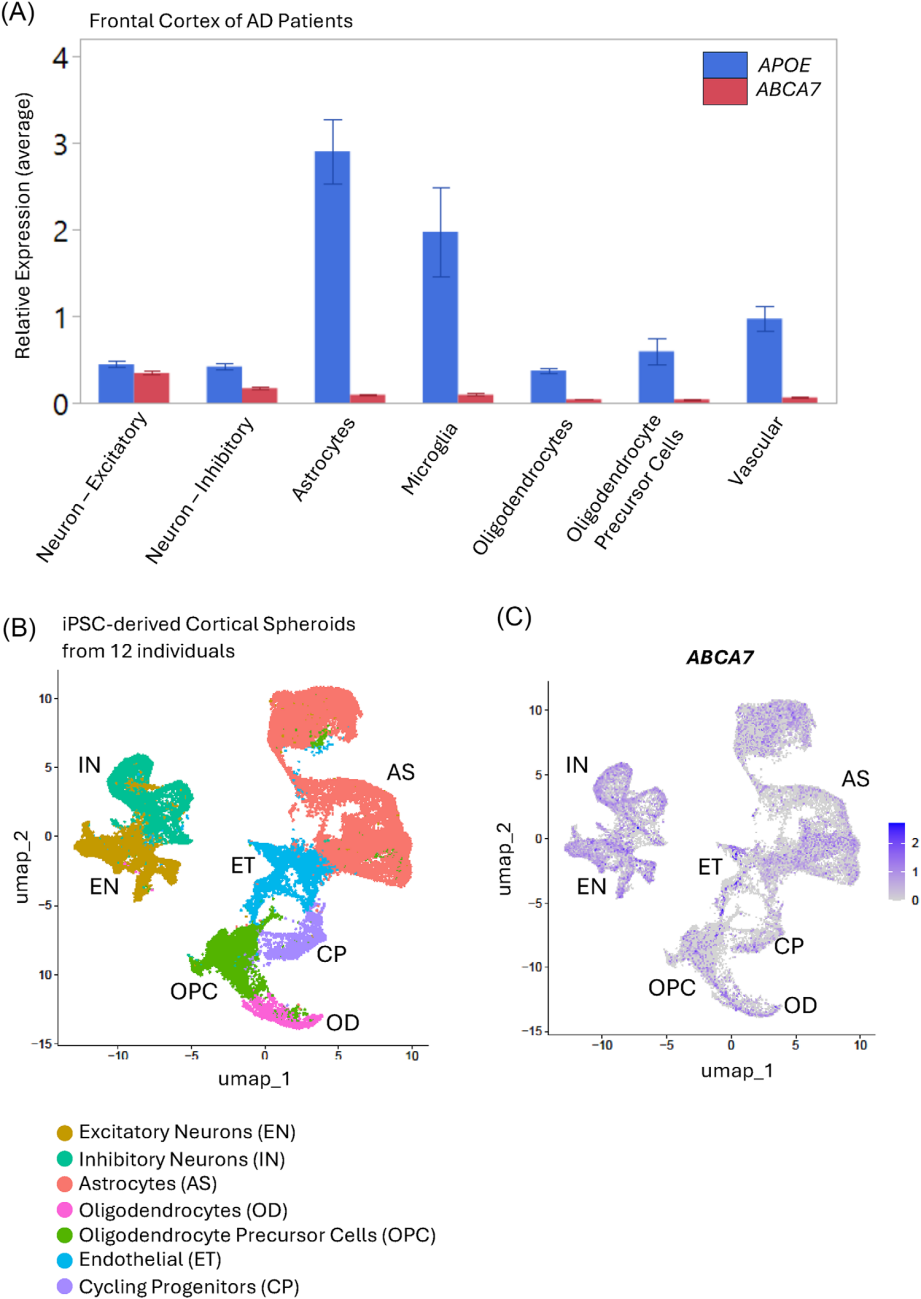
*ABCA7* is expressed brain‐wide at relatively low levels, with the highest expression in neurons. A, *ABCA7* (red) and *APOE* (blue) expression level comparison across cell types from single‐nucleus RNA sequencing of the frontal cortex of AD patients. B, UMAP of single‐cell RNAseq of iPSC‐derived cortical spheroids from 12 individuals, colored based on cell‐type cluster (left) or *ABCA7* expression levels (right). ABCA7, ATP binding cassette subfamily A member 7; AD, Alzheimer's disease; *APOE*, apolipoprotein E; iPSC, induced pluripotent stem cell; UMAP, uniform manifold approximation and projection.

Previous studies have reported that *ABCA7* transcriptional expression is generally low across various brain cell types but is higher in neurons compared to non‐neuronal glial cells.^26^, ^27^, ^28^ Consistent with previous reports, our single‐nuclei RNA sequencing (snRNAseq) data from human frontal cortex showed that *ABCA7* expression was highest in neurons (Figure 4A).^29^ Figure 4A shows a comparison to *APOE* expression levels, known to be minimal in neurons, revealing the extent of expression of *ABCA7*. To support this finding, we also analyzed *ABCA7* expression levels in iPSC‐derived neural spheroids, which contain neurons, astrocytes, and oligodendrocytes (Figure 4B and C).^30^ The data belong to a joint analysis of neural spheroids derived from four iPSC lines from individuals of varied genetic ancestries (African, Amerindian, and European) and AD status (Figure S8A and B in supporting information). The analysis indicated that neurons in the iPSC‐derived spheroid exhibited the highest *ABCA7* transcript levels across the global ancestry and AD status.

### 3.4 ABCA7 truncation alters lipid metabolism in neurons

**FIGURE 5:**
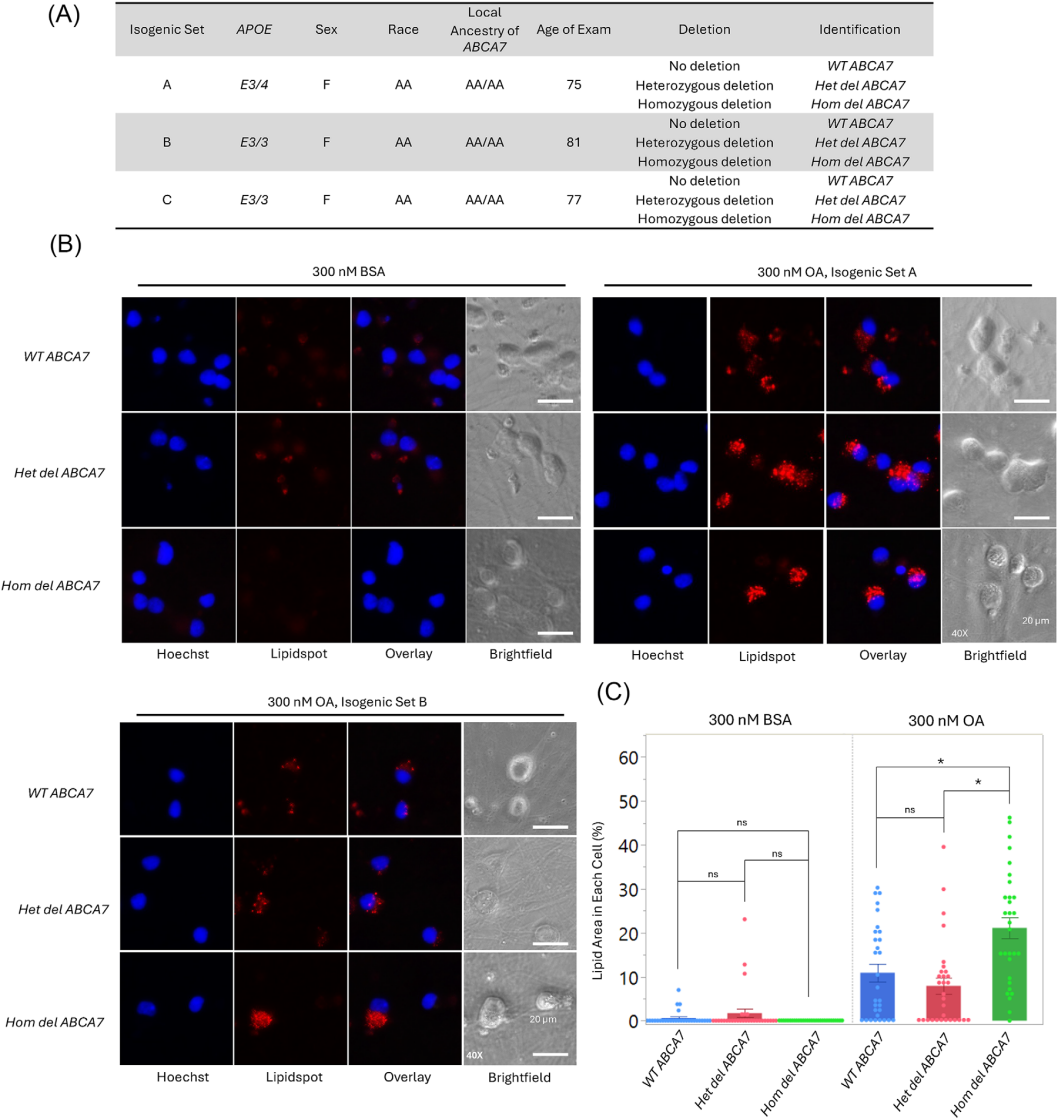
Increased neuronal lipid droplet accumulation in homozygous carriers of the *ABCA7* deletion. A, Table of isogenic iPSC lines derived from unaffected individuals with an AA background. CRISPR‐based genome editing was used to introduce the AA‐specific *ABCA7* deletion in homozygous or heterozygous states. B, Representative ICC images (isogenic set A and B) showing LD accumulation in isogenic neurons carrying WT *ABCA7*, heterozygous *ABCA7* deletion, or homozygous *ABCA7* deletion after treatment with 300 nM BSA or OA. Fluorescence images were obtained on a Keyence BZ‐X800 microscope using a 40× objective and were cropped for presentation. Scale bar, 20 µm. C, Quantitation of lipid area (%) in individual isogenic neurons stained with LipidSpot. The lipid area from individual neurons per well was averaged. These experiments were performed with technical triplicates. The dots overlaid on each bar represent the individual data points. Data are presented as mean ± standard error of the mean. *P < 0.01, ns, non‐significant (one‐way analysis of variance followed by Tukey *t* test for post hoc analysis). AA, African ancestry; ABCA7, ATP binding cassette subfamily A member 7; BSA, bovine serum albumin; ICC, immunocytochemistry; iPSC, induced pluripotent stem cell; LD, lipid droplet; OA, oleic acid; WT, wild type.

The relatively high expression of *ABCA7* in neurons prompted us to analyze the effect of the *ABCA7* truncation in this cell type. Thus, we generated isogenic iPSC lines containing either WT *ABCA7* or lines heterozygous or homozygous for the *ABCA7* deletion introduced into these lines by CRISPR‐based genome editing. The *ABCA7* deletion was introduced into iPSCs derived from three independent, cognitively unimpaired, non‐deletion carrier individuals of AA, resulting in three sets of isogenic *ABCA7* lines.^31^ Each isogenic set included a CRISPR‐unedited control line, a heterozygous *ABCA7* deletion line, and a homozygous *ABCA7* deletion line (Figure 5A). These lines were validated for pluripotency, and genomic stability by G‐banding karyotyping, and Sanger sequencing confirmed the genotypes and screened for potential CRISPR off‐target effects.^22^ After validation, these lines were induced to become human glutamatergic neurons via transgenic expression of *NGN2*.^32^

To test whether the *ABCA7* deletion affected LD accumulation in neurons, as observed in HepG2 cells, we exposed the nine isogenic *ABCA7* neuronal lines to either 300nM BSA (vehicle control) or 300nM OA and quantified LD accumulation (Figure 5B). LipidSpot quantification revealed that neurons exposed to BSA did not exhibit significant LD accumulation (Figure 5C). However, OA exposure induced LD formation across all isogenic neuronal sets (Figure 5C). Analysis of the three independent isogenic sets (A, B, and C) revealed a consistent pattern, in which neurons with homozygous *ABCA7* deletion exhibited a significant increase in LD accumulation compared to both WT *ABCA7* and heterozygous *ABCA7* deletion neurons.

### 3.5 ABCA7 truncation reduces mitochondrial action potential in neurons

**FIGURE 6:**
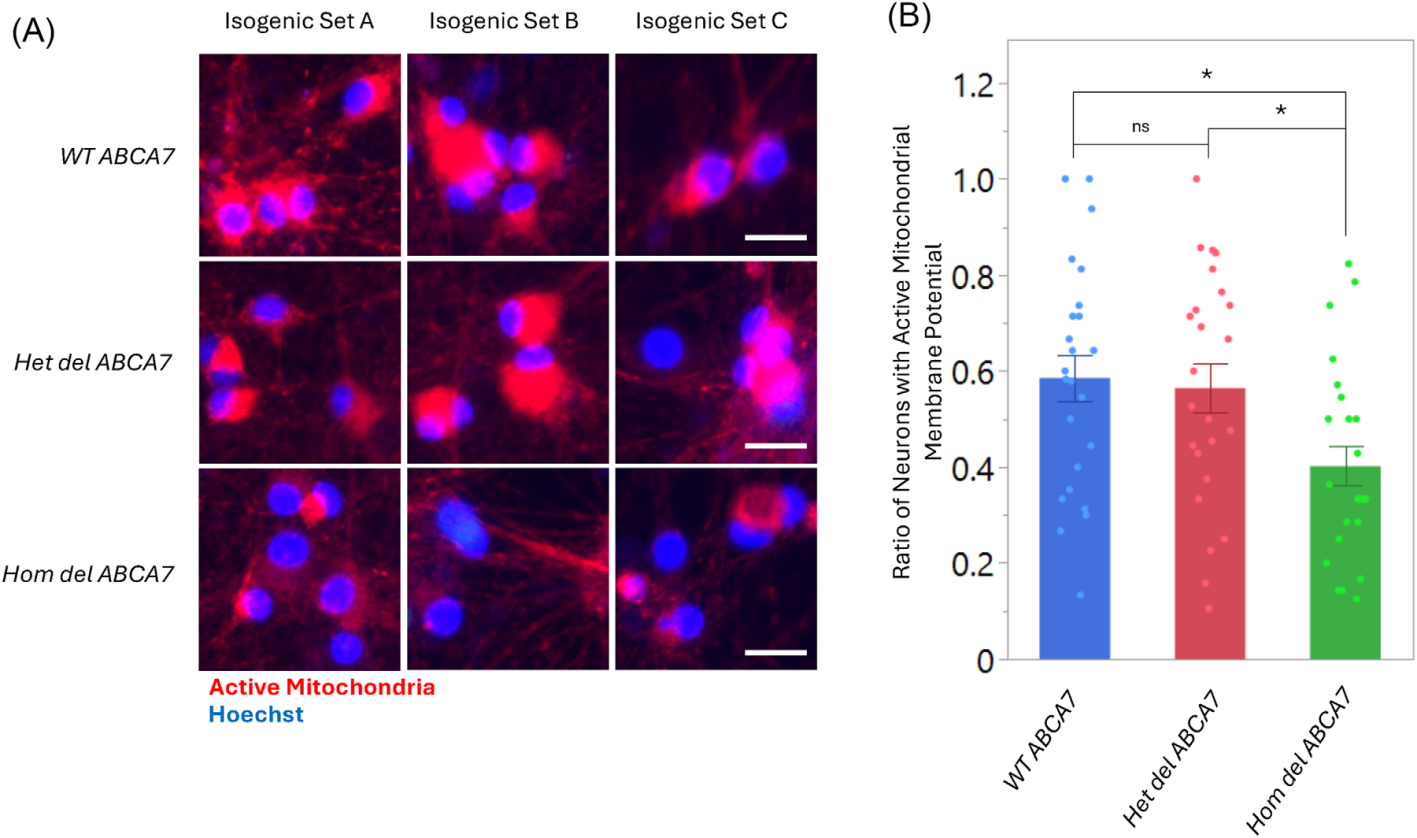
Decreased active mitochondrial action potential in a homozygous carrier of the *ABCA7* deletion. A, Mitochondrial health in isogenic WT, heterozygous deletion, and homozygous deletion *ABCA7* cells, assessed using a mitochondrial health kit. Red indicates active mitochondrial membrane potential; nuclei are stained with Hoechst. Fluorescence images were obtained on a Keyence BZ‐X800 microscope using a 40× objective and were cropped for presentation. Scale bar, 20 µm. B, Quantitation of the intensity of active mitochondrial potential in individual isogenic neurons. These experiments were performed with technical replicates. The dots overlaid on each bar represent the individual data points. Data are presented as mean ± standard error of the mean. *P < 0.01, ns, non‐significant (one‐way analysis of variance followed by Tukey *t* test for post hoc analysis). ABCA7, ATP binding cassette subfamily A member 7; WT, wild type.

Excessive lipids promote lipotoxicity and oxidative stress in mitochondria and alter mitochondrial membrane composition and integrity.^33^, ^34^ These studies have demonstrated that loss‐of‐function *ABCA7* variants negatively affect mitochondria in neurons as a potential AD‐associated phenotype.^33^, ^34^ To test whether the AA‐specific deletion *ABCA7* affects mitochondrial function, we measured changes in the mitochondrial membrane potential using a reagent that accumulates in active mitochondria of aged neurons carrying WT *ABCA7*, heterozygous deletion *ABCA7*, or homozygous deletion *ABCA7* (Figure 6A). The result showed that Hom del *ABCA7* significantly decreased the active mitochondria compared to WT *ABCA7* iNeurons (Figure 6B). This analysis supports the impact of the AA‐specific del *ABCA7* in AD‐associated phenotypes.

## 4 DISCUSSION

This study sheds light on the functional consequences of the AA‐associated 44‐bp deletion in *ABCA7*, a variant strongly associated with increased AD risk. Unlike most nonsense mutations in *ABCA7*, which produce truncated proteins that are mislocalized or degraded,^18^ we find that the truncated ABCA7 protein resulting from the 44‐bp deletion is both stable and correctly trafficked, residing mostly at the ER membrane and to a lesser degree at the plasma membrane in HEK cells. These findings challenge the widely held assumption that AD risk associated with ABCA7 truncations operates solely through haploinsufficiency and opens the door to alternative pathogenic mechanisms. The presence of a membrane‐localized truncated ABCA7 protein raises the possibility of dominant–negative effects, in which the mutant protein may interfere with the WT allele's function in heterozygous carriers. This model aligns with clinical observations that heterozygous and, in some cases, homozygous *ABCA7* 44‐bp deletion carriers often present with similar disease severity and age of onset.^35^

One significant finding from our study is the demonstration that the 44‐bp deletion in *ABCA7* increases LD accumulation. This defect observed in liver‐derived HepG2 cells was consistent with the observation of significantly elevated LD levels in iPSC‐derived human neurons homozygous for the deletion. These results are in line with prior work in animal models, including *Drosophila*, showing that *ABCA7* loss leads to lipid accumulation,^36^ and in mice, where *ABCA7* knockout impairs lipid metabolism and endolysosomal function.^18^, ^37^

LDs are cellular organelles essential for lipid storage, metabolism, signaling, and protection against lipotoxicity.^38^ The significance of LDs in AD has been extensively documented. For instance, microglial LD accumulation, associated with pathogenic *APOE* variants, induces tau phosphorylation and subsequent neurotoxicity.^11^ Furthermore, apoE is transported to astrocyte LDs, where it influences triglyceride saturation and the size of the LDs.^39^ Although neurons typically possess a lower intrinsic capacity for LD storage compared to glia,^12^, ^40^ maintaining lipid homeostasis is crucial for synaptic health and resistance to metabolic stress, particularly during aging. Excessive lipid accumulation in neurons impairs mitochondrial function through disrupted β‑oxidation and lipid peroxidation, which compromises mitochondrial membrane integrity, reduces membrane potential, and can trigger mitophagy.^41^, ^42^, ^43^ This mitochondrial dysfunction further disrupts neuronal homeostasis by limiting energy availability, ultimately leading to synaptic dysfunction and neuronal apoptosis, which can contribute to the progression of AD pathology.^44^, ^45^ As such, our finding that the *ABCA7* deletion promotes lipid accumulation and mitochondrial dysfunction provides evidence that this deletion can contribute to the progression of AD pathology.

The observation that *ABCA7* is expressed at the highest levels in neurons in the brain—based on both snRNAseq of human brain tissue and analysis of iPSC‐derived neural spheroids—underscores the potential for a cell‐autonomous, neuron‐specific role of ABCA7 in lipid regulation. While earlier studies suggested inconsistent cell‐specific expression, indicating slightly higher levels in astrocytes than neurons^28^—likely due to low overall *ABCA7* expression and experimental limitations, more recent data consistently show that neurons exhibit higher *ABCA7* levels than glial cell types.^27^, ^34^, ^46^ To further assess *ABCA7* expression at the protein level in a cell type–specific context, we attempted to measure endogenous ABCA7 protein expression in iPSCs and iNeurons; however, no detectable signal was observed using WB, ICC, or high‐sensitivity mass spectrometry–based proteomics for the inherently low expression level of ABCA7 protein. Thus, the transcriptional data provide the most meaningful evidence and support studying neurons for ABCA7 functional studies.

Our identification of a functional defect caused by the *ABCA7* deletion suggests that this variant may influence AD risk through disrupted lipid processing pathways. This finding was further supported by studies using isogenic human iPSC‐derived neurons, in which we observed increased LD accumulation and decreased mitochondrial action potential in homozygous deletion cells. Our findings align with recent reports demonstrating a critical role for ABCA7 in neuronal lipid homeostasis.^14^ These mechanisms may be especially relevant in the context of *APOE* variants, which also disrupt lipid handling and synergize with ABCA7 dysfunction.^12^ The convergence of these pathways points toward a lipid‐centered model of AD risk and suggests that impaired lipid metabolism in neurons is the pathological mechanism that leads to the increased risk seen in *ABCA7* 44‐bp deletion carriers.

An intriguing aspect of our findings is that neurons with heterozygous *ABCA7* deletion did not exhibit significant lipid or mitochondrial phenotypes, despite the clear clinical relevance of heterozygous deletion carriers. Several factors may account for this discrepancy. Although iPSC‐based models provide important advantages for investigating AD‐related mechanisms, iPSC‐derived neurons do not fully capture the complexity of the human brain.^47^ First, these neurons reflect an early developmental stage and may lack aging‐associated stressors necessary to unmask subtle defects that arise in heterozygous deletion states. Second, our model evaluated neurons in monoculture and therefore does not capture cell–cell interactions with astrocytes and microglia that occur in the brain. Third, essential features of the in vivo microenvironment, including chronic aging‐related influences and multicellular context, are absent in this model system. In addition, because the *ABCA7* deletion acts as a risk factor rather than a fully penetrant causal mutation, these limitations are likely to reduce the sensitivity of the in vitro system to detect modest pathogenic effects associated with heterozygous deletion. Notably, similar observations have been reported in other iPSC‐based disease models. For example, in iPSC‐derived hematopoietic models of RPA1 E240K, cellular phenotypes such as telomere shortening and impaired differentiation were observed only in homozygous mutant lines, whereas heterozygous lines appeared phenotypically normal in vitro, despite clinical evidence that heterozygous carriers exhibit disease manifestations.^48^ This highlights a broader limitation of iPSC models in capturing subtle, dose‐sensitive effects associated with heterozygous risk variants.

Our recent epidemiological studies indicate that the *ABCA7* deletion reduces the age of onset in individuals carrying the *APOE* ε3/ε4 allele compared to those without the deletion or with other *APOE* genotypes.^21^ Indeed, a previous report suggested that *ABCA7–APOE* interactions may influence memory function.^49^ apoE plays a crucial role in lipidation and lipid transport for maintaining lipid homeostasis in the brain.^50^ apoE localizes to the surface of LD in astrocytes, regulating LD size, turnover, and sensitivity to peroxidation.^39^ *APOE* ε4 has been shown to dysregulate LD dynamics, potentially contributing to its strong risk for AD. Therefore, the truncated ABCA7 as a consequence of the deletion may further hinder the transport of lipids seen in *APOE* ε4 carriers, leading to an earlier age of onset. Further studies investigating the functional interaction between *ABCA7* deletions and *APOE* alleles are necessary to clarify this relationship and could provide greater clarity into the pathological mechanism of *APOE* ε4.

Taken together, these findings support a model in which the 44‐bp deletion in *ABCA7* increases AD risk through impaired regulation of lipid homeostasis, with loss of *ABCA7* function leading to LD accumulation and mitochondrial dysfunction in neurons. Whether this occurs via haploinsufficiency, dominant–negative effects, or context‐dependent loss of function remains to be fully elucidated. Importantly, our study is the first to demonstrate that the truncated ABCA7 protein resulting from the 44‐bp deletion is stable and traffics to the ER and plasma membrane, providing critical mechanistic insight into how this variant may disrupt lipid metabolism.

These results not only lay the groundwork for understanding how ABCA7‐mediated lipid dysregulation and mitochondrial dysfunction contribute to AD pathogenesis but also highlight the importance of ancestry‐informed functional genomics in uncovering population‐specific mechanisms by which *ABCA7* variants drive neurodegeneration.

## CONFLICT OF INTEREST STATEMENT

The authors declare no conflicts of interest. Author disclosures are available in the supporting information.

## CONSENT STATEMENT

No human data were derived and, therefore, consent was not necessary.

## Supporting information



Supporting Information

Supporting Information
