# Supplementary material for: Disrupted lipid homeostasis as a pathogenic mechanism in ABCA7‐associated Alzheimer's disease risk
Source: Alzheimers Dement. 2026 Mar 30;22(4):e71312. doi: 10.1002/alz.71312 (PMC13140508; doi:10.1002/alz.71312)
Supplement: Supplementary file 1 — Supporting Information [file ALZ-22-e71312-s002.docx]

**
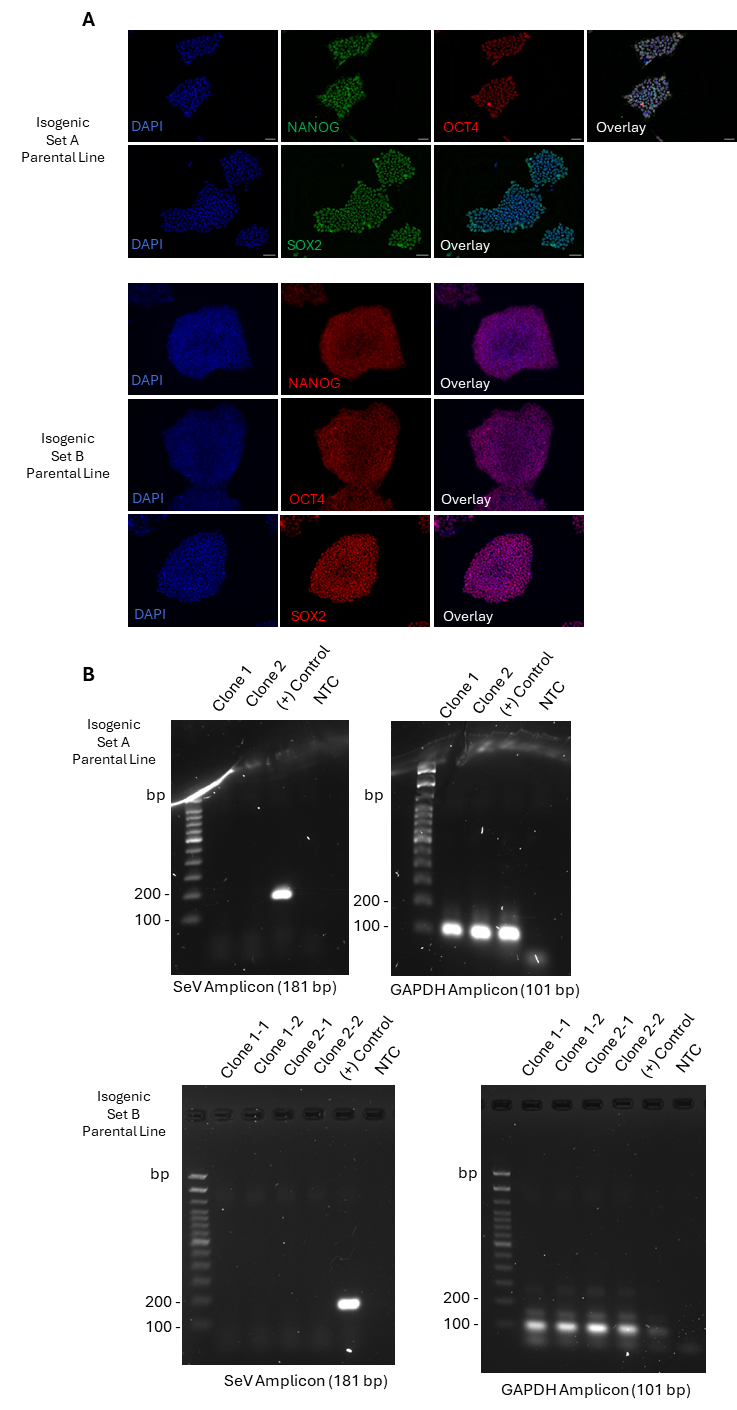
**

**Supplementary Figure 1.** Characterization of pluripotency and Sendai virus clearance in iPSC lines. (A) Pluripotency of the parental iPSC lines A and B was assessed by immunostaining for NANOG, SOX2, and OCT4 in accordance with ISSCR guidelines. (B) Clearance of reprogramming vectors was confirmed by PCR analysis, demonstrating the absence of Sendai viral sequences in both iPSC lines.

**
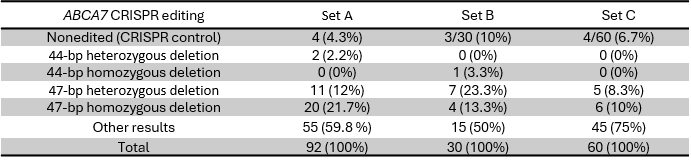
**

CRISPR control


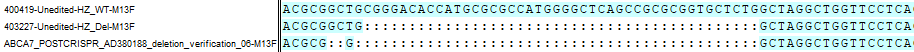


44-bp deletion

44-bp deletion *ABCA7*


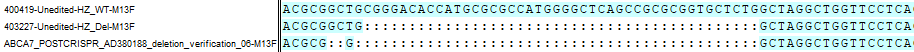

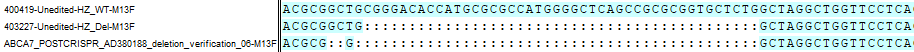


47-bp deletion

47-bp deletion *ABCA7*


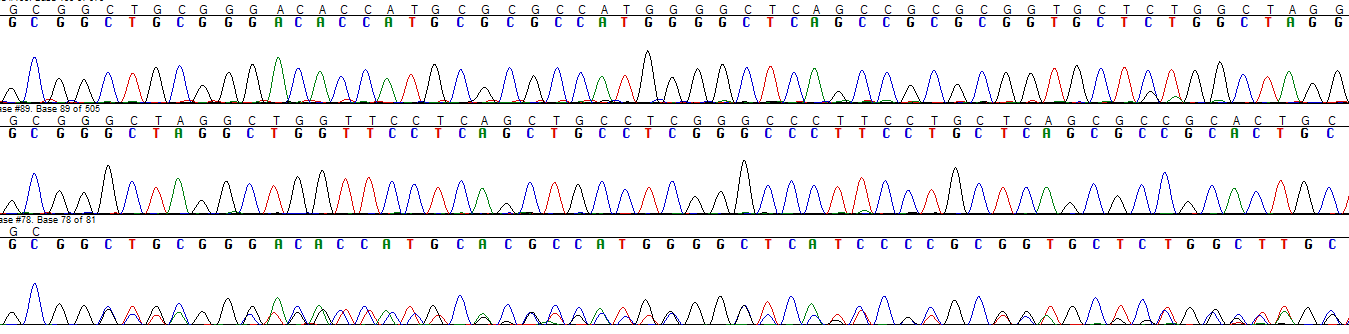

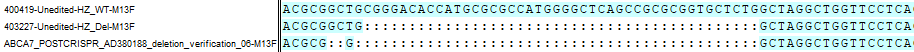

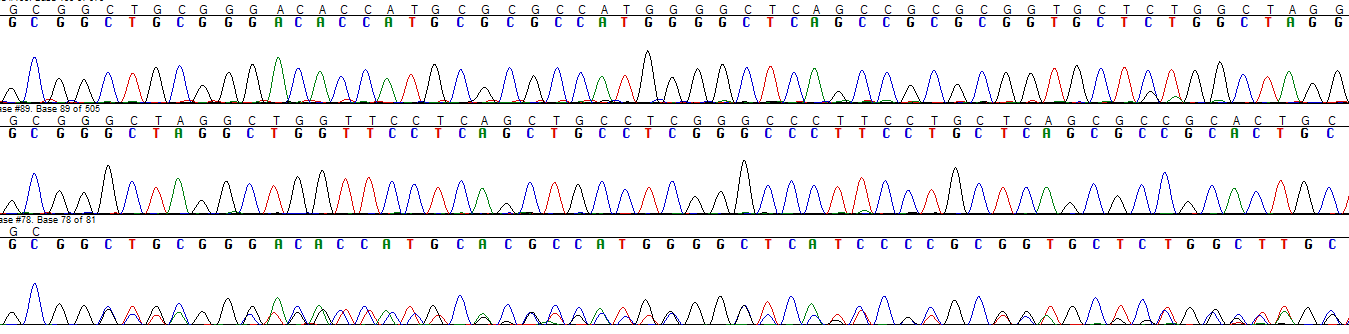

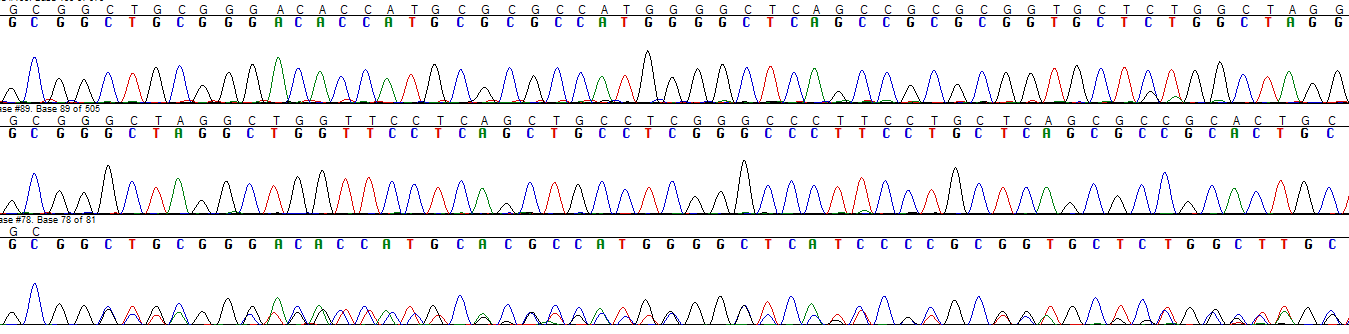


47-bp Homozygous deletion *ABCA7*

47-bp Heterozygous deletion *ABCA7*

**B**

**A**

**C**

p.Arg578Alafs

44-bp del ABCA7

**Arg(R)578Ala(A)**

ARLVPQLPRALPAQRRTAGSGAQAGRHPPLQPPGRGLPVLGSLRGGHGDPELPAQRLLLPRQPGCGLRRPGLLLPLPALRAVCGLAGPAARGWPRGREPAVARGLRLRLREPGSAGGAGRGRAVAQRGHPAYGRRLQPGPGLWPSAAGRGALRPRHLVPGSCVPRPVRDP*

1 2

p.Lue577Alafs

47-bp del ABCA7

**Leu(L)577Ala(A)**

ARLVPQLPRALPAQRRTAGSGAQAGRHPPLQPPGRGLPVLGSLRGGHGDPELPAQRLLLPRQPGCGLRRPGLLLPLPALRAVCGLAGPAARGWPRGREPAVARGLRLRLREPGSAGGAGRGRAVAQRGHPAYGRRLQPGPGLWPSAAGRGALRPRHLVPGSCVPRPVRDP*

1 2

**Supplementary Figure 2.** Development of CRISPR isogenic *ABCA7* iPSC lines. (A) CRISPR control, 44-bp heterozygous and homozygous *ABCA7* deletion clones were identified. Fewer 44-bp *ABCA7* deletion clones were obtained compared to 47-bp *ABCA7* deletion clones. (B) Sanger sequencing chromatogram confirmed the 44 and 47-bp deletion in *ABCA7* using CRISPR-Cas9 gene editing. (C) A schematic protein diagram of the predicted proteins encoded by the 44 and 47-bp deletions at the plasma membrane. The predicted protein from the 47-bp deletion loses another amino acid due to the three additional nucleotides removed, but it maintains the same remaining protein sequences, including the early termination, as the 44-bp *ABCA7* deletion.

**
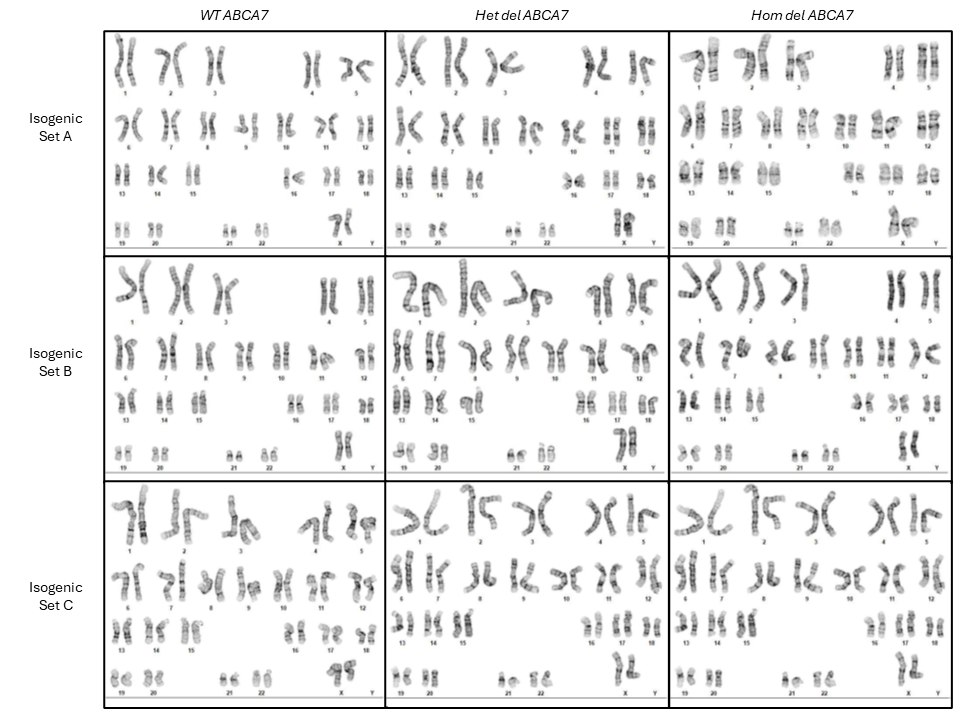
**

**Supplementary Figure 3.** Normal karyotypes were observed for all isogenic iPSC lines.

**
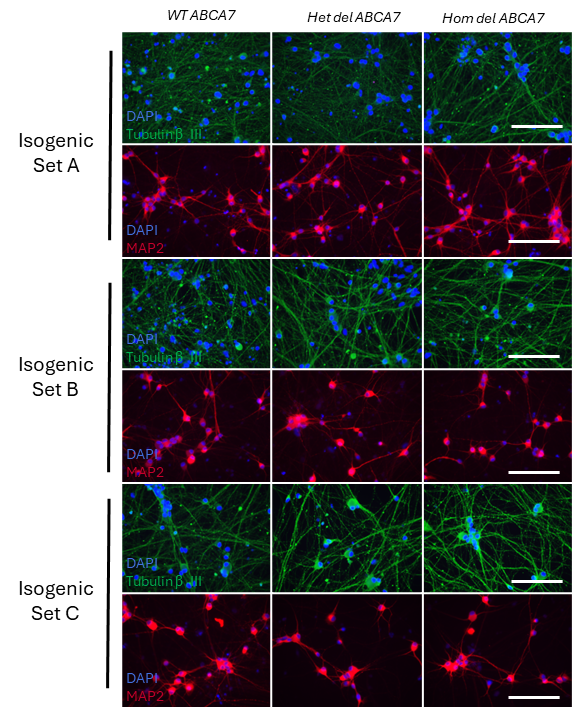
**

**Supplementary Figure 4.** Neuronal differentiation (iNeuron) using a transdifferentiation approach. iPSC-derived neurons were stained with DAPI, Tubulin β III (green), and MAP2 (red) to identify the quality of neuronal differentiation. The transdifferentiation approach produced a homogenous neuronal density in all the isogenic lines. Fluorescence images were obtained on a Keyence BZ-X800 microscope using a 40× objective. Scale bar, 20 µm.

**
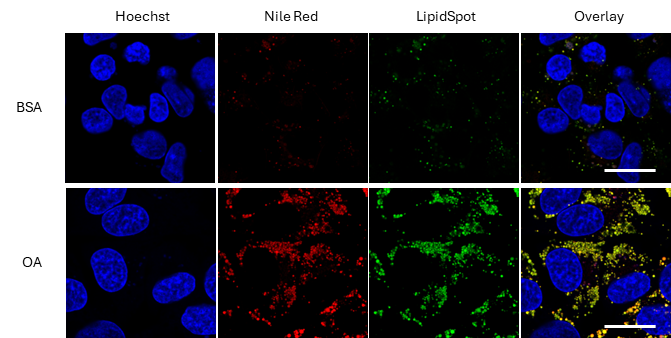
**

**Supplementary Figure 5.** Validation of LipidSpot labeling for lipid droplet detection. HepG2 cells exposed to 300 nM BSA or oleic acid (OA) were stained with Nile Red and LipidSpot to assess LD content. Nuclei were counterstained with Hoechst. Confocal images were acquired using a ZEISS LSM 980 equipped with Airyscan 2 and a 63× oil-immersion objective with 3× optical zoom. Scale bar, 16.7 µm.

**
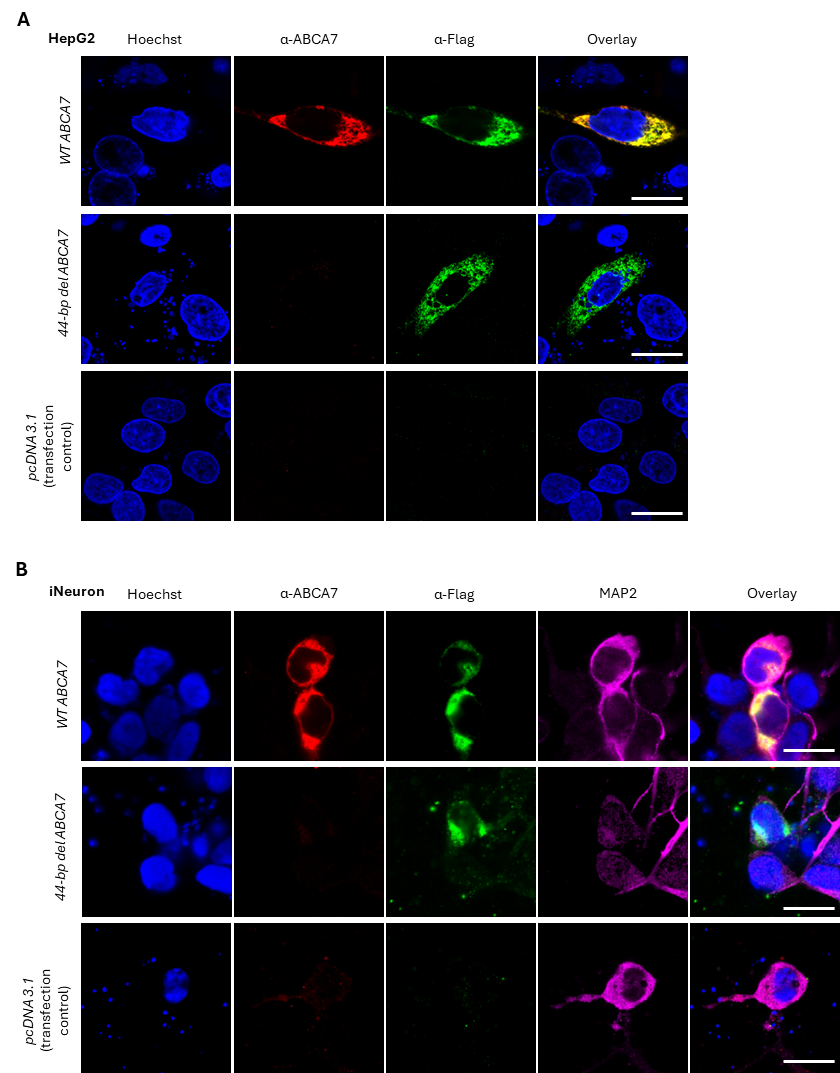
**

**Supplementary Figure 6.** Truncated ABCA7 is stable and localizes at the ER membrane and plasma membrane in HepG2 and early iNeurons. Immunocytochemistry (ICC) of (A) HepG2 cells and (B) iNeurons transfected with either WT *ABCA7,* truncated *ABCA7,* or *pcDNA3.1* (empty vector, transfection control) probed with Hoechst (nucleus marker), anti-Flag, anti-ABCA7, or anti-MAP2 (neuronal marker) antibodies. The faint ABCA7 signal observed in the pCDNA3.1 control likely reflects either microscope overexposure or non-specific antibody binding, rather than true ABCA7 expression. Confocal images were collected on a ZEISS LSM 980 equipped with Airyscan 2 using a 63× oil-immersion objective and 3× optical zoom. Scale bar, 16.7 µm.


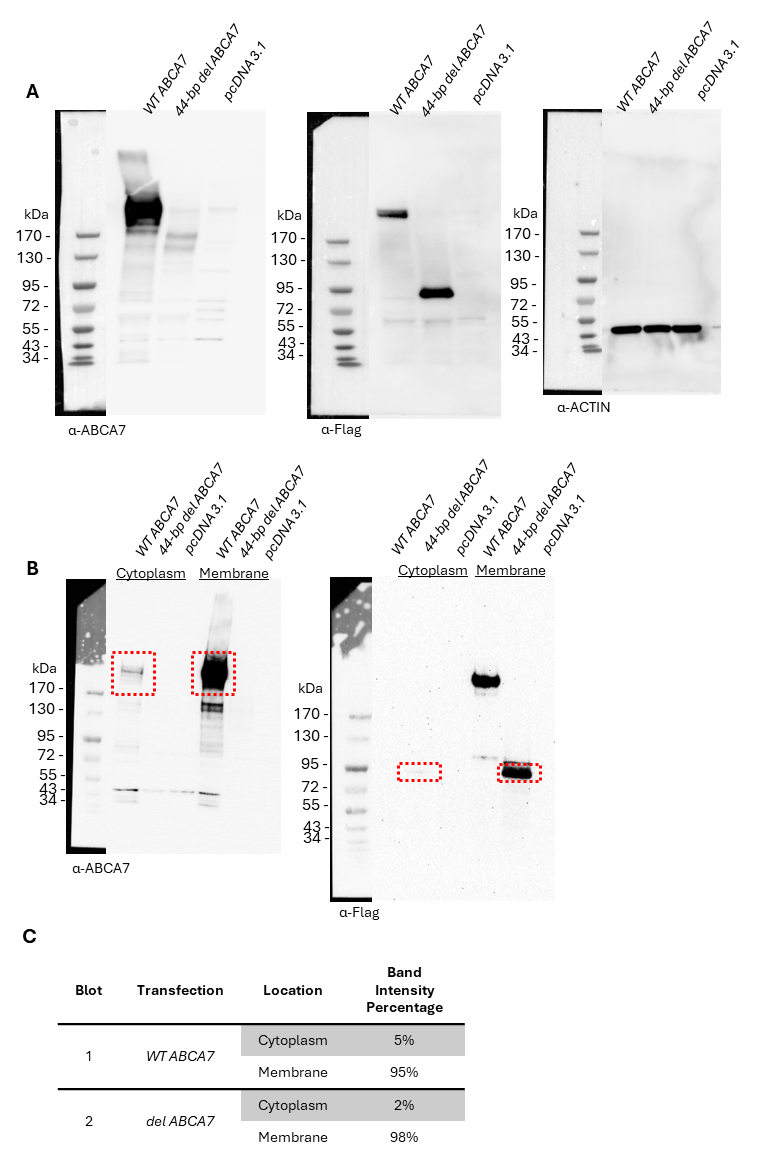


**Supplementary Figure 7.** Truncated ABCA7 is stable and predominantly localizes to the plasma membrane. Full Western blot images corresponding to Figure 2 are shown in panels (A) and (B). (C) Densitometric analysis of the bands delineated by the red dashed boxes in panel (B) was performed using ImageJ (FIJI) to quantify the relative abundance and subcellular localization of ABCA7. Western blot for subcellular localization was performed once; no statistical analysis was done due to limited replicates.


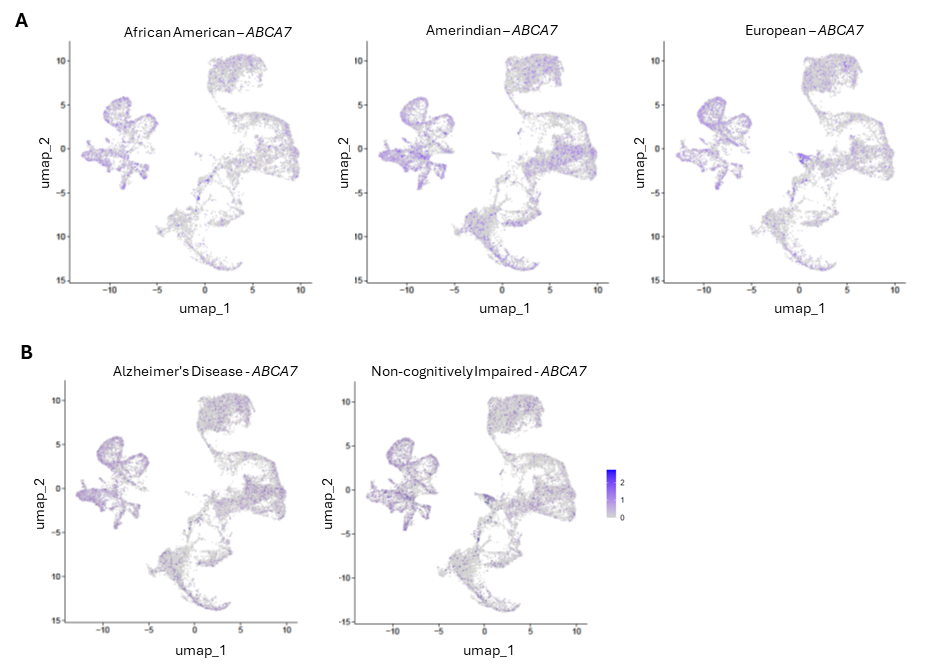


**Supplementary Figure 8.** UMAP of single-cell RNA-sequencing profiles from iPSC-derived cortical spheroids generated from 12 individuals. Cell clusters are colored by *ABCA7* expression and grouped by (A) local ancestry and (B) disease status.
